# Modeling Selective Pressures on Phytoplankton in the Global Ocean

**DOI:** 10.1371/journal.pone.0009569

**Published:** 2010-03-10

**Authors:** Jason G. Bragg, Stephanie Dutkiewicz, Oliver Jahn, Michael J. Follows, Sallie W. Chisholm

**Affiliations:** 1 Department of Civil and Environmental Engineering, Massachusetts Institute of Technology, Cambridge, Massachusetts, United States of America; 2 Department of Earth, Atmospheric and Planetary Sciences, Massachusetts Institute of Technology, Cambridge, Massachusetts, United States of America; 3 Department of Biology, Massachusetts Institute of Technology, Cambridge, Massachusetts, United States of America; American Museum of Natural History, United States of America

## Abstract

Our view of marine microbes is transforming, as culture-independent methods facilitate rapid characterization of microbial diversity. It is difficult to assimilate this information into our understanding of marine microbe ecology and evolution, because their distributions, traits, and genomes are shaped by forces that are complex and dynamic. Here we incorporate diverse forces—physical, biogeochemical, ecological, and mutational—into a global ocean model to study selective pressures on a simple trait in a widely distributed lineage of picophytoplankton: the nitrogen use abilities of *Synechococcus* and *Prochlorococcus* cyanobacteria. Some *Prochlorococcus* ecotypes have lost the ability to use nitrate, whereas their close relatives, marine *Synechococcus*, typically retain it. We impose mutations for the loss of nitrogen use abilities in modeled picophytoplankton, and ask: in which parts of the ocean are mutants most disadvantaged by losing the ability to use nitrate, and in which parts are they least disadvantaged? Our model predicts that this selective disadvantage is smallest for picophytoplankton that live in tropical regions where *Prochlorococcus* are abundant in the real ocean. Conversely, the selective disadvantage of losing the ability to use nitrate is larger for modeled picophytoplankton that live at higher latitudes, where *Synechococcus* are abundant. In regions where we expect *Prochlorococcus* and *Synechococcus* populations to cycle seasonally in the real ocean, we find that model ecotypes with seasonal population dynamics similar to *Prochlorococcus* are less disadvantaged by losing the ability to use nitrate than model ecotypes with seasonal population dynamics similar to *Synechococcus*. The model predictions for the selective advantage associated with nitrate use are broadly consistent with the distribution of this ability among marine picocyanobacteria, and at finer scales, can provide insights into interactions between temporally varying ocean processes and selective pressures that may be difficult or impossible to study by other means. More generally, and perhaps more importantly, this study introduces an approach for testing hypotheses about the processes that underlie genetic variation among marine microbes, embedded in the dynamic physical, chemical, and biological forces that generate and shape this diversity.

## Introduction

The genomes, functional traits, and distributions of marine microbes are shaped by a complex mixture of dynamic physical, biogeochemical, and biological forces. Physical transport processes strongly regulate nutrient re-supply to different ocean habitats. Microbes assimilate these nutrients, reducing their concentrations in seawater, leading to competition for growth substrates [1]. These processes influence microbial growth and population dynamics, in combination with other ecological processes, such as predation [2]. Over time, the abilities of microbes to acquire and assimilate specific nutrients change by evolution: mutation can cause the loss or gain of nutrient use abilities, and the frequencies of individuals in populations with different nutrient use abilities can change through selection [3] or other processes such as genetic drift or hitchhiking [4]. It is not possible to examine how interactions between these temporally varying physical, ecological and mutational processes influence the traits of marine microbes by studying them in isolation from each other. It is therefore a major challenge to develop approaches that are capable of explicitly representing all of these dynamic processes in a common modeling framework.

**Figure 1:**
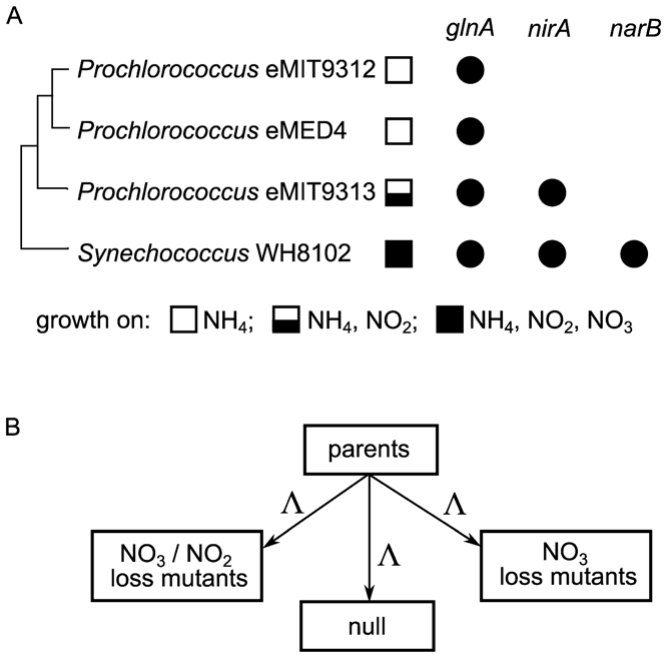
Diagrams illustrating our modeling approach. (A) Marine *Synechococcus* and *Prochlorococcus* isolates vary in their abilities to grow on NH_4_, NO_2_ and NO_3_ [6], [8], as indicated by the boxes next to the strain names and key below [6], [8]. These nitrogen use abilities are reflected in the genomes of the isolates, including the presence of genes needed for assimilating NH_4_ (*glnA*), NO_2_ (*nirA*) and NO_3_ (*narB*) (solid dots indicate the presence of a particular gene [13]–[15]). (B) All picophytoplankton in the model initially can use NO_3_, NO_2_ and NH_4_. After three years, three different types of mutations occur, each at the same rate, Λ (see Text S1). The different types of mutants produced are (i) mutants that cannot use NO_3_; (ii) mutants that cannot use NO_2_ or NO_3_; (iii) null mutants that retain the ability to use NO_3_, NO_2_ and NH_4_.

We begin to address this challenge by incorporating these processes into a global numerical simulation to study the selective pressures on marine picophytoplankton for specific nitrogen use abilities. Marine picocyanobacteria *Prochlorococcus* and *Synechococcus* are dominant phytoplankton in tropical and subtropical ocean ecosystems [5]. These genera consist of ‘ecotypes’ that are specialized for ocean habitats that differ in irradiance, temperature, and nutrient availability. In broad terms, the nitrogen use abilities of *Prochlorococcus* and *Synechococcus* ecotypes are related to the availability of different nitrogen sources in their habitats. Many, but not all, *Synechococcus* isolates can use nitrate (NO_3_), nitrite (NO_2_) and ammonium (NH_4_) as sources of nitrogen [6], [7]. In contrast, many *Prochlorococcus* ecotypes appear to have lost the ability to use nitrate. All *Prochlorococcus* isolates that have been tested cannot use nitrate [6], [8] (Fig. 1A), though there are wild *Prochlorococcus* cells that contain the nitrate reductase gene (*narB*), transcribe it, and use nitrate [9], [10].

The inability of many *Prochlorococcus* to use nitrate is consistent with their dominance of stratified surface waters that are relatively nitrate-poor and ammonium-rich [11], [12]. In open ocean regions, nitrite is often relatively abundant in the lower euphotic zone, but less abundant nearer the surface. Correspondingly, many *Prochlorococcus* ecotypes that are adapted to high light also lack the ability to use nitrite [6], [8], whereas studies of cultured cells have revealed that some low light adapted *Prochlorococcus* ecotypes retain this ability [6]. The inability to grow on nitrate or nitrite is associated with the loss of genes necessary for using these compounds, such as nitrate and nitrite reductase [13]–[15] (Fig. 1A). Despite these broad qualitative observations, the abundances of *Prochlorococcus* and *Synechococcus* do not show strong statistical associations with the distribution of inorganic nitrogen compounds [16], [17], though nitrate availability can explain some variation in *Prochlorococcus* community composition [18]. Gaps therefore exist in our understanding of how the availability of these nitrogen compounds influences the distribution and evolution of *Prochlorococcus* and *Synechococcus*. Here we provide new insights into this by modeling selective pressures that might have attended the evolution of these traits historically, or which contribute to the maintenance of these traits in the modern ocean.

## Results

### Assaying Selective Pressures on Phytoplankton in an Ocean Model

To examine selective pressures on modeled picophytoplankton nitrogen use abilities, we simulated picophytoplankton growth in the global ocean, introduced mutants lacking the ability to use specific nitrogen compounds, and studied the fate of these mutants. This approach for assaying selective pressures was applied within an existing ocean ecosystem model [19] (see Text S1), which previously provided a good characterization of the distribution and abundance of major phytoplankton functional groups, and in particular the picocyanobacteria, in the global ocean. The model generates a community of phytoplankton, consisting of many “large” and “small” phytoplankton types, whose functional traits are assigned at random from realistic ranges of values. The large phytoplankton represent taxa that are fast-growing under nutrient replete conditions, including diatoms, and the small phytoplankton can grow at relatively low levels of nutrient availability and light, and represent picophytoplankton [19]. The ecosystem model is integrated forward, and ecological interactions among modeled phytoplankton and grazers determine the distribution of different phytoplankton types [19]. In the present study, we initialized simulations with 15 types of picophytoplankton and 18 types of large phytoplankton. After three years had elapsed, subtropical regions were largely dominated by picophytoplankton, and higher latitudes were largely dominated by the large phytoplankton groups [19]. We then began to apply mutations to the picophytoplankton for the loss of specific nitrogen use abilities.

All modeled phytoplankton could initially use nitrate, nitrite and ammonium. The use of nitrate and nitrite was repressed by abundant ammonium, consistent with observations [20]–[22] and previous models [23]–[25]. When picophytoplankton grew in the model, a small proportion of divisions (∼10^−8^) produced mutants, of three different types. The first mutant type was unable to use nitrate (‘NO_3_ loss mutant’), and the second was unable to use either nitrate or nitrite (‘NO_3_/NO_2_ loss mutant’) (Fig. 1B). We might think of these mutants as lacking functional nitrate reductase (*narB*) and nitrite reductase (*nirA*) genes, respectively, as the former is essential for assimilating nitrate, and the latter for assimilating both nitrite and nitrate [22]. The third type of mutant was identical to its parent in all respects (a ‘null mutant’), including the ability to use NO_3_, NO_2_ and NH_4_ (Fig. 1B). Since each kind of mutant was produced at an identical rate, we assessed the disadvantage associated with losing specific nitrogen use abilities in different locations by considering the relative abundances of the different mutants (related approaches have a long history in population genetics, *e.g.* [26]). That is, we asked: in which parts of the ocean is it strongly disadvantageous to lose specific nitrogen use abilities, and in which parts is it less disadvantageous?

Our approach examines the selective pressures attending the evolution of nitrogen use abilities in different regions of the ocean, by studying the fate of rare mutants with different nutrient use traits. While we derived and parameterized the model with the goal of representing mutation and selection in ways that reflect real-world processes, it is important to note that our model also omits important evolutionary processes, such as frequency dependent selection, due to the short time scales of the simulations. Also, we did not provide any advantages to mutants that lack the ability to use nitrate or nitrite [27]. While it is plausible such advantages exist, they are poorly understood, and we do not have any estimates of their magnitude. These simulations therefore consider disadvantages associated with losing these nutrient use abilities in isolation from any potential tradeoffs associated with loss-of-function mutations, and offer insights into pressures that help shape the course of evolution, rather than attempting to model the course of evolution in every detail.

### Biogeography Influences the Selective Consequences of Losing N Use Abilities

**Figure 2:**
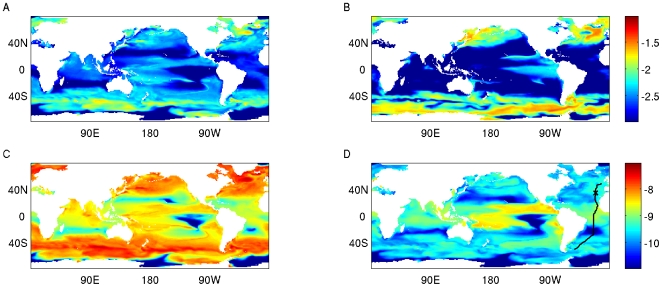
The distribution of phytoplankton biomass and accumulation of NO_3_/NO_2_ loss mutants in modeled surface waters. The global distribution of phytoplankton (phosphorus biomass [log_10_(µM P)], 0 to 10 m, annual average) is plotted for (A) picophytoplankton and (B) large phytoplankton. The distribution of mutant picophytoplankton (phosphorus biomass [log_10_(µM P)], 0 to 10 m, annual average) is plotted for (C) null mutants and (D) NO_3_/NO_2_ loss mutants. Data shown are for the fifth year of one integration, out of an ensemble of ten integrations that were initialized with different, randomly generated, phytoplankton communities. In panel (D), the cruise track of Atlantic Meridional Transect 13 (AMT 13) is indicated with a black line, and the location 35°N, 22°W is indicated with a cross.

We first consider the consequences of losing the ability to use NO_3_ and NO_2_ in surface (upper 10 m) waters across the global ocean (Fig. 2). Picophytoplankton parent populations were dominant in tropical and subtropical surface waters. At higher latitudes, picophytoplankton reached considerable abundances (Fig. 2A), but large phytoplankton tended to dominate (Fig. 2B). Mutant picophytoplankton accumulated to different abundances in surface waters across the range of the parent picophytoplankton (Fig. 2 C,D). The NO_3_/NO_2_ loss mutants reached substantial abundances in tropical ocean regions, where their abundances were similar to those of null mutants in some locations (Fig. 2C, D). In contrast, at higher latitudes, null mutants accumulated to much greater abundances than the NO_3_/NO_2_ loss mutants, indicating that the NO_3_/NO_2_ loss mutants were disadvantaged in these regions (Fig. 2C, D).

These results indicate that the selective pressure to retain nitrate and nitrite use abilities was weakest in tropical regions of the modeled ocean, including oligotrophic tropical ocean regions where *Prochlorococcus* is the dominant pico-phytoplankter in the real ocean [5], [16], [17], [28]. Among the different ocean basins, the accumulation of NO_3_/NO_2_ loss mutants was greater in the tropical Pacific than in tropical regions of the Indian and Atlantic (Fig. 2D). This reflects limitation by iron in the modeled tropical Pacific [29], which diminishes the selective consequences associated with losing the ability to use NO_3_ and NO_2_. The reason is, when modeled phytoplankton are strongly limited by iron, such that their growth is relatively insensitive to nitrogen availability (including the availability of NO_3_ and NO_2_), there is no growth disadvantage associated with lacking the ability to use NO_3_ and NO_2_. This model result highlights the notion that complex interactions between the abundances of different nutrients influence the selective pressures on specific nutrient use abilities, and suggests the hypothesis that genes encoding nitrate reducing proteins will be less prevalent in *Prochlorococcus* living in iron or phosphorus limited regimes – a hypothesis that can be tested, now that numerous *Prochlorococcus narB* sequences are available for primer design [10].

At higher latitudes, where larger quantities of inorganic nitrogen are available as NO_3_ or NO_2_ (see Text S1), the selective pressure against the NO_3_/NO_2_ loss mutants was greater. The key results described above were consistent across an ensemble of 10 simulations that had different randomly generated phytoplankton communities, and in simulations using different parameter values for the mutation rate, and some biogeochemical transformations of nitrogen (see Text S1).

**Figure 3:**
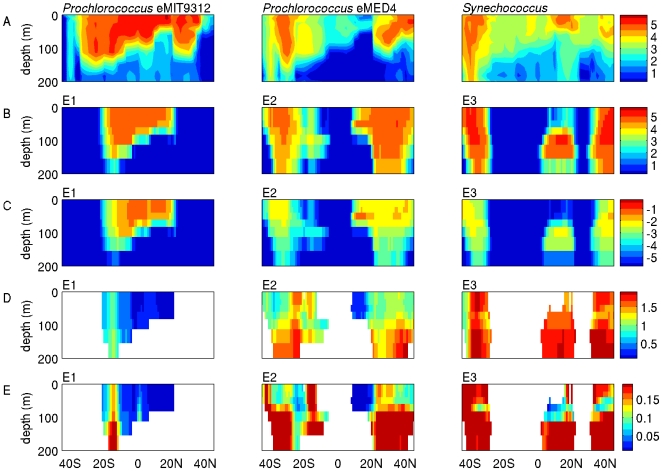
Observations and model predictions along a latitudinal transect. The distribution of *Prochlorococcus* and *Synechococcus* picocyanobacteria along AMT13, and model predictions for the accumulation of NO_3_/NO_2_ loss mutants in their model analogs. (A) The distribution of two *Prochlorococcus* ecotypes and *Synechococcus* along AMT13 [log_10_(cells ml^−1^)]. (B) The abundance of three model phytoplankton types along AMT13 that have similar distributions to real-world picocyanobacteria (converted to log_10_(cells ml^−1^) assuming 1 fg P cell^−1^). (C) The abundance of NO_3_/NO_2_ loss mutants of these modeled ecotypes (converted to log_10_(cells ml^−1^) assuming 1 fg P cell^−1^). (D, E) Indices of the disadvantage associated with losing the ability to use nitrate (D) and nitrite (E) for each ecotype. The disadvantage associated with losing the ability to use nitrate is calculated as log_10_[ (null)/(NO_3_ loss mutant)], and the disadvantage associated with losing the ability to use nitrite is calculated as log_10_[(NO_3_ loss mutant)/(NO_3_/NO_2_ loss mutant)]. These indices are plotted for each ecotype in locations where the parent is abundant (greater than 10^−3^ times its maximum value).

We next consider a latitudinal transect in the Atlantic ocean [30] (AMT 13; Fig. 2D) where data on abundances of *Prochlorococcus* ecotypes and *Synechococcus* are available [16]. Model runs often contained ‘analogs’ of picocyanobacterial groups, in terms of their biogeography and functional traits [19]. Here, model phytoplankton E1 was most abundant in tropical surface waters, displaying a biogeography that is similar to that of real-world *Prochlorococcus* ecotype eMIT9312 (Fig. 3A, B). The model phytoplankton E2 was most abundant at slightly higher latitudes, in surface waters of the subtropics, similar to *Prochlorococcus* ecotype eMED4 (Fig. 3A, B). Modeled phytoplankton E3 was most abundant at greater latitudes still, above 40°, where observed abundances of *Prochlorococcus* decline, and *Synechococcus* are the dominant picocyanobacteria [16], [28] (Fig. 3A, B). We will therefore consider phytoplankton types E1, E2 and E3 to be ecological analogs of *Prochlorococcus* eMIT9312, *Prochlorococcus* eMED4 and *Synechococcus*, respectively.

Among these ecological analogs of picocyanobacteria, there was variation in the selective consequences associated with losing NO_3_ and NO_2_ use abilities. The NO_3_/NO_2_ loss mutants of modeled ecotype E1 accumulated to relatively great abundances (Fig. 3C), and the disadvantage associated with losing the ability to use NO_3_ and NO_2_ was relatively small (Fig. 3D and E, respectively), consistent with the inability of cultured representatives of *Prochlorococcus* eMIT9312 to use nitrate or nitrite [6]. In contrast, the NO_3_/NO_2_ loss mutants of ecotype E3 reached smaller abundances, and the disadvantage associated with losing NO_3_ and NO_2_ use abilities was relatively large, consistent with the retention of nitrate and nitrite use abilities by many marine *Synechococcus* [6]. These results are likely related to the relatively low availability of NO_3_ and NO_2_ in regions where E1 was most abundant, and greater availability of NO_3_ near the surface in regions where E3 was abundant, though there is not a simple correspondence between the selective consequences of losing these nitrogen use abilities and the annual average concentrations of inorganic nitrogen compounds (see Text S1). Model ecotype E2 was intermediate between E1 and E3 in terms of the disadvantage associated with losing the use of NO_3_ and NO_2_. In particular, this is interesting in regions along the transect where ecotypes E2 and E3 were both abundant (Fig. 3B). For example, near the surface at latitudes 35°–45°, both E2 and E3 had substantial annual average abundances (Fig. 3B), but the loss of NO_3_ and NO_2_ use abilities had greater deleterious consequences for E3 than for E2 (Fig. 3D and E). To better understand why model ecotypes E2 and E3 living in the same location experience different selective consequences from the loss of nitrogen use abilities, we next consider seasonal patterns in the abundance of ecotype E2 and E3 parent and mutant populations at a single location.

### Population Dynamics Influence the Selective Consequences of Losing N Use Abilities

**Figure 4:**
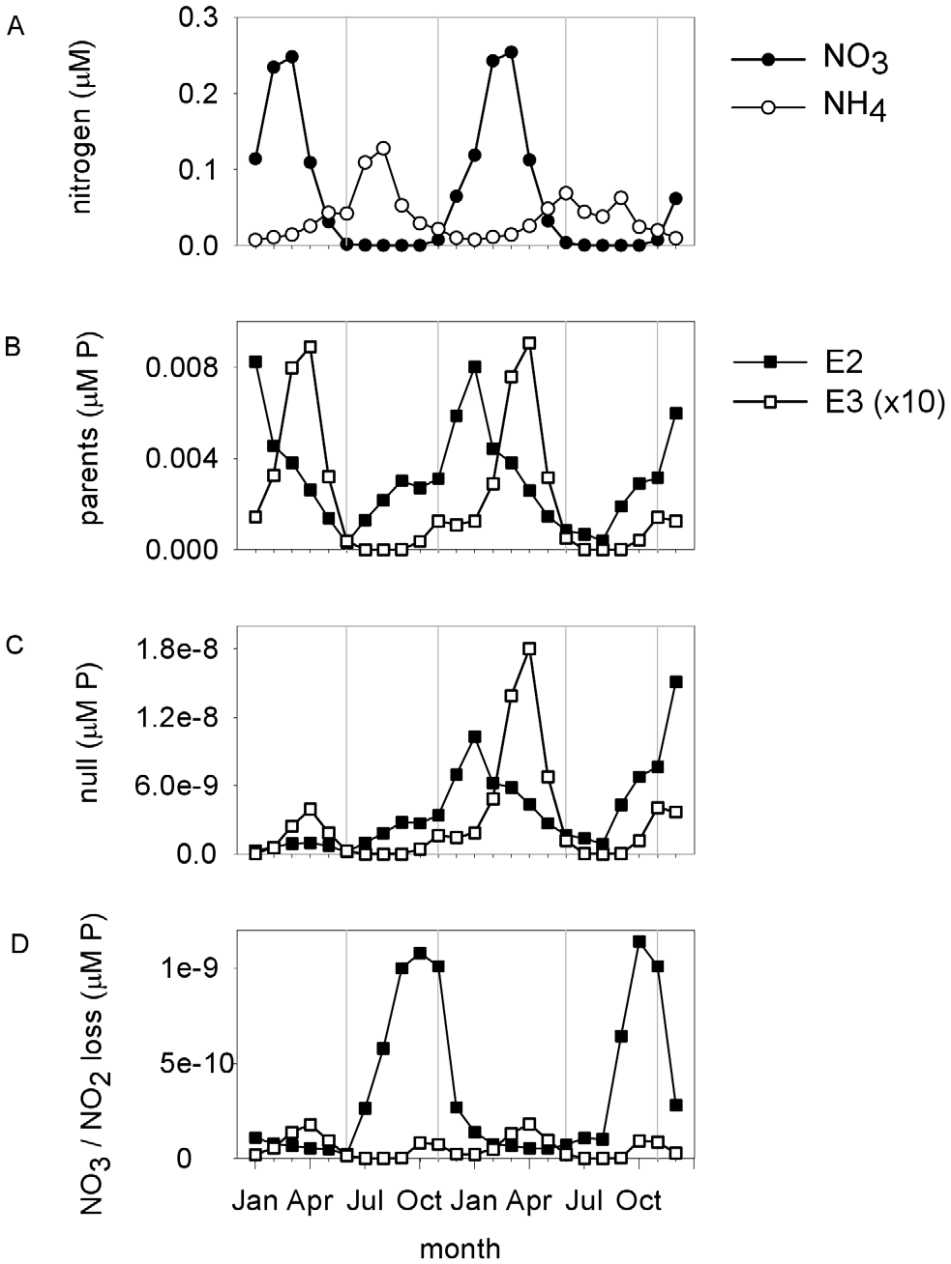
Seasonal patterns in the abundances of nutrients and phytoplankton. Seasonal patterns in the availability of (A) ammonium and nitrate, and the abundance of (B) modeled ecotypes E2 and E3 (ecological analogs of *Prochlorococcus* eMED4 and *Synechococcus*, respectively), and their (C) null mutants and (D) NO_3_/NO_2_ loss mutants, at the location 35°N, 22°W, near the surface.

In ocean regions like the North Atlantic near Bermuda, with alternating periods of stratification and deep mixing of the water column, *Prochlorococcus* and *Synechococcus* populations vary seasonally [31]. *Prochlorococcus* reaches its greatest abundances in autumn following summer stratification, while *Synechococcus* reaches its greatest abundances following deep mixing in spring, when the nitracline is shallow [31]. Parent populations of our modeled ecotypes E2 and E3 exhibited such cyclical population dynamics in surface waters at 35°N (Fig. 4). E2 became abundant during the summer when nitrate was scarce, but its abundance dropped sharply around January (Fig. 4A, B), similar to observations for *Prochlorococcus* near Bermuda [31]. E3 increased in abundance during autumn when nitrate was entrained in the upper euphotic zone, and dropped sharply as the water column became stratified in summer (Fig. 4A, B), similar to *Synechococcus* [31]. Modeled ecotype E2 had a higher optimum temperature for growth and a slightly lower nutrient half-saturation constant than E3, which may explain its dominance during summer stratification of the water column.

The seasonal population dynamics of these modeled ecotypes influenced the selective consequences of losing the ability to use nitrate and nitrite. When the population of ecotype E2 increased rapidly around June, for example, both its null mutants and NO_3_/NO_2_ loss mutants also increased in abundance (Fig. 4C, D). Around December, when the availability of nitrate increased rapidly, the abundance of NO_3_/NO_2_ loss mutants of ecotype E2 dropped sharply. In contrast, parent and null mutant populations of modeled ecotype E3 increased rapidly at times of the year when nitrate was highly abundant. The NO_3_/NO_2_ loss mutants were competitively disadvantaged under these circumstances, and their population increased much less rapidly (Fig. 4). This shows how, in this location, losing the ability to use NO_3_ and NO_2_ had larger deleterious consequences for modeled ecotype E3 than for E2, because E3 is dominant during a time of year when NO_3_ was a major source of nitrogen.

## Discussion

We have developed and applied an approach for studying selective pressures on phytoplankton functional traits in a model of the global ocean. Here we consider selective pressures on nitrogen use abilities in modeled pico-phytoplankton that are differentiated on the basis of other physiological traits, including preferred levels of temperature and irradiance. At the global scale, we find that losing the ability to use nitrate is least deleterious in modeled phytoplankton with biogeographical distributions similar to those of *Prochlorococcus*. Thus our model results are broadly consistent with the observation that *Prochlorococcus* have a greater tendency than *Synechococcus* to be unable to use nitrate [6].

In addition to conforming to this broad generalization, our simulations make detailed spatial and temporal predictions that offer insights into dynamic processes that are difficult to study by empirical means, and which are too complex to deduce in the absence of quantitative tools. For instance, our analysis of the abundances of mutant populations over seasonal cycles shows how two phytoplankton ecotypes that live in similar ocean regions but which have different temporal population dynamics, might face different selection pressures for the retention of nitrogen use genes.

These results highlight the importance of studying the physical, biogeochemical, ecological and evolutionary processes affecting marine microbes in a common setting. The modeling approach presented here is applicable to a range of different questions about the evolution of microbial traits and microbial diversity in the sea, and will offer valuable opportunities to study global marine microbial communities in the context of the diverse and dynamic forces that act upon them.

## Supporting Information

Text S1 Supporting information on methods and results.
(1.31 MB PDF)
