# Supplementary material for: Modeling Selective Pressures on Phytoplankton in the Global Ocean
Source: PLoS One. 2010 Mar 10;5(3):e9569. doi: 10.1371/journal.pone.0009569 (PMC2835739; doi:10.1371/journal.pone.0009569)
Supplement: Text S1 — Supporting information on methods and results. (1.31 MB PDF) [file pone.0009569.s001.pdf]

Supporting Information

Methods

1. Global ocean ecosystem model

The ocean ecosystem model used in this study has been described previously by Follows et al. [1]. Changes in the concentration of the *i*th nutrient (N_i), *j*th phytoplankton group (P_j), *k*th zooplankton group (Z_k) and pools of particulate organic matter (POM_i) and dissolved organic matter (DOM_i) are modeled with the following equations:

$$\frac{\partial N_i}{\partial t} + \nabla \cdot (\boldsymbol{u} N_i) = \nabla \cdot (\kappa \nabla N_i) - \sum_j \left[\mu_j P_j R_{ij}\right] + S_{N_i} \quad \text{(SE1)}$$

$$\frac{\partial P_j}{\partial t} + \nabla \cdot (\boldsymbol{u} P_j) = \nabla \cdot (\kappa \nabla P_j) - \frac{\partial (w_j^P P_j)}{\partial z} - m_j^P P_j - \sum_k [g_{jk} P_j Z_{k,i=1}] + \mu_j P_j \quad \text{(SE2)}$$

$$\frac{\partial Z_{ki}}{\partial t} + \nabla \cdot (\boldsymbol{u} Z_{ki}) = \nabla \cdot (\kappa \nabla Z_{ki}) - m_k^Z Z_{ki} + Z_{k,i=1} \sum_j [\zeta_{jk} g_{jk} P_j R_{ij}]. \quad \text{(SE3)}$$

$$\frac{\partial POM_i}{\partial t} + \nabla \cdot (\boldsymbol{u} POM_i) = \nabla \cdot (\kappa \nabla POM_i) - r_{POM_i} POM_i - \frac{\partial (w_{POM} POM_i)}{\partial z} + S_{POM_i} \quad \text{(SE4)}$$

$$\frac{\partial DOM_i}{\partial t} + \nabla \cdot (\boldsymbol{u} DOM_i) = \nabla \cdot (\kappa \nabla DOM_i) - r_{DOM_i} DOM_i + S_{DOM_i} \quad \text{(SE5)}$$

The parameters used in these equations are defined below (Table S1) and by Follows et al. [1] and Dutkiewicz et al. [2]. Inorganic nitrogen, phosphorus, iron and silicon nutrients (N_i) are taken up by phytoplankton. The rates of uptake are determined by the abundance (P_j, expressed in terms of phosphorus biomass), growth rate (μ_j) and elemental ratios (R_{ij}) of the phytoplankton. The inorganic nutrients are supplied by different processes that are represented here by S_i and can include remineralization, supplies from external sources, and abiotic transformations. Phytoplankton growth rates (μ_j) are determined by irradiance, nutrient availability, and temperature (see below). Phytoplankton sink through the water column according to a sinking rate, w_j^P, and are lost by mortality m_j^P and grazing by zooplankton. The rate of grazing by the *k*th zooplankton on the *j*th phytoplankton is represented by g_{jk}, which in turn is given by the function $g_{jk} = g_{mjk} \frac{\eta_{jk}}{A_k} \frac{A_k}{A_k + k_k^P}$. Here g_{mjk} is the maximum rate at which the *k*th zooplankton can graze the *j*th phytoplankton per unit of phytoplankton biomass, η_{jk} is the palatability of the *j*th phytoplankton to the *k*th zooplankton, A_k is the sum of phytoplankton weighted by their palatability, $A_k = \sum_j \left[\eta_{jk} P_j\right]$, and k_k^P is the value of A_k at which grazing by the *k*th zooplankton reaches half its maximum rate. Zooplankton grow according to their rate of grazing, g_{jk}, and feeding efficiency, ζ_{jk}, and are lost by mortality, m_k^Z.

The terms S_{POM_i} and S_{DOM_i} represent the rates of supply of POM_i and DOM_i (respectively) from the decomposition of biomass, excretion, and inefficient feeding by

zooplankton on phytoplankton. Fixed proportions of decomposing and excreted material (f_{POM}), and of grazed but unconsumed material (g_{POM}), pass to the POM pool, and the remaining proportions pass to the DOM pool. The terms r_{POM_i} and r_{DOM_i} represent the rate of loss of POM_i and DOM_i (respectively) by remineralization to inorganic compounds.

The constants $\boldsymbol{u}$ and κ represent advection and diffusion (respectively) in the model ocean and were provided by the ECCO ("Estimating the Circulation and Climate of the Ocean") state estimates [3]. These equations were integrated in a 1° X 1° resolution ocean circulation model with 23 vertical layers [4]. The advection scheme used for these simulations was the 'superbee' second order flux limiter [5]. We also applied an additional flux limiter to prevent spurious negative concentrations that occur due to the enforcement that mixing occur along isopycnal surfaces.

These simulations were performed with 33 different types of phytoplankton, and 2 different types of zooplankton. The physiological properties and nutrient requirements of different phytoplankton groups were assigned randomly, subject to some simple taxonomic and allometric constraints, using the approach of Follows et al. [1]. In each integration, we initialized 15 'small' phytoplankton types and 18 'large' phytoplankton types. Small phytoplankton have slower maximum growth rates than large phytoplankton, but are able to grow at lower levels of light and nutrient availability (Fig. S1). Initial concentrations of nutrients and grazers were determined using previous simulations [1]. An ensemble of 10 integrations was performed, using different randomly generated phytoplankton communities.

2. Phytoplankton growth and nutrient use

Phytoplankton growth rates, μ_j, are determined by multiplying the maximum growth rate μ_j for a phytoplankton group by three factors that represent the degree of limitation by temperature (γ_j^T), irradiance (γ_j^I), and nutrient availability (γ_j^N), such that $\mu_j = \mu_{mj}\gamma_j^T\gamma_j^I\gamma_j^N$. Values of γ_j^T, γ_j^I and γ_j^N are each scaled to have a value between 0 and 1, and are dimensionless. The response of growth to local temperature (T) is represented by the function $\gamma_j^T = \frac{1}{\tau_1}(A^T \exp(-B(T-T_O)^C) - \tau_2)$, where A, B, C and T_O regulate the shape of the dependence of growth on temperature, and τ_1 and τ_2 are used to normalize γ_j^T to a maximum value (see Fig. S1A). The response of growth to local irradiance (I) is represented by the function $\gamma_j^I = \frac{1}{F_m}(1-\exp(-k_{PAR}I))\exp(-k_{inhib}I)$, where k_{PAR} controls the saturation of growth as a function of irradiance, k_{inhib} controls the inhibition of growth by high irradiance, and F_m is a factor that is used to normalize γ_j^I to a maximum value of 1 (Fig. S1B). The degree of nutrient limitation is determined by the ambient nutrient concentration that supports the lowest rate of growth, or $\gamma_j^I = \min(N_i^L)$.

For iron, phosphorus and silica (diatoms only), $N_i^L = \dfrac{N_i}{N_i + k_{N_{ij}}}$, where $k_{N_{ij}}$ is the concentration of the *i*th nutrient at which the *j*th phytoplankton reaches half its maximum growth rate (Fig. S1C). Nitrogen is available in three inorganic forms, NO_3, NO_2 and NH_4. The use of NO_3 and NO_2 are repressed when NH_4 is available. This is represented in the model using the expression

$$N_N^L = \frac{NO_3 + NO_2}{NO_3 + NO_2 + k_{N_{iNO}}}\exp(-\psi NH_4) + \frac{NH_4}{NH_4 + k_{N_{iNH4}}}.$$

During the course of the simulations, we introduce phytoplankton mutants (see below) that are unable to use NO_3, but retain the ability to use NO_2 and NH_4. For these mutants, we use the expression

$$N_N^L = \frac{NO_2}{NO_2 + k_{N_{iNO}}}\exp(-\psi NH_4) + \frac{NH_4}{NH_4 + k_{N_{iNH4}}}.$$

Other mutants lose the ability to use both NO_3 and NO_2, but retain the ability to use NH_4. For these mutants, we represent limitation by nitrogen as

$$N_N^L = \frac{NH_4}{NH_4 + k_{N_{iNH4}}}.$$

3. Mutation

In the present study, we introduce mutations for the loss (and gain) of specific nitrogen use abilities. We apply these mutations to each of the 15 ‘small’ phytoplankton types, beginning after the third year of the simulation. At this point the model has settled into a relatively steady annually repeating cycle of phytoplankton abundances. Initially, all biomass in a specific phytoplankton type can use NO_3, NO_2 and NH_4 as nitrogen sources. We refer to this biomass as comprising the ‘parent’ population. The process of mutation (see Fig. 1B) is simulated by transferring biomass between the parent population and an additional tracer that represents the biomass of ‘mutant’ phytoplankton that have identical properties to their parent, except for a specific difference that corresponds to the mutated phenotype.

The changes in the concentration of mutant (P_M) and parent (P_P) biomass are modeled as follows:

$$\frac{\partial P_P}{\partial t} + \nabla\cdot(\boldsymbol{u}P_P) = \nabla\cdot(\kappa\nabla P_P) - L_P + \mu_P P_P - \Lambda + \Gamma$$

$$\frac{\partial P_M}{\partial t} + \nabla\cdot(\boldsymbol{u}P_M) = \nabla\cdot(\kappa\nabla P_M) - L_M + \mu_M P_M + \Lambda - \Gamma$$

where the growth of a specific parent population by cell division is described by $\mu_P P_P$, and for a specific mutant by $\mu_M P_M$, as in Eq. SE2 and described in Section 2 of the Methods. A fraction, Λ_f, of cell divisions result in a mutation (such as the loss of the ability to use nitrate) producing mutant biomass (P_M) at a rate of

$$\Lambda = \Lambda_f \log_2(e)\mu_P P_P,$$

where $\log_2(e)$ adjusts the growth rate to cell divisions per day. We also allow a fraction, Γ_f, of cell divisions of mutant biomass to result in mutation back to the parent phenotype. This results in the production of parent biomass at a rate of

$$\Gamma = \Gamma_f \log_2(e)\mu_M P_M .$$

L_P and L_M represent the change in the concentration of parent and mutant phytoplankton (respectively) per unit biomass from sinking, mortality and grazing (as in Eq. SE2), or

$$L_P = \frac{\partial(w_P^P P_P)}{\partial z} + m_P^P P_P + \sum_k [g_{Pk} P_P Z_{k,i=1}] \text{, and}$$

$$L_M = \frac{\partial(w_M^P P_M)}{\partial z} + m_M^P P_M + \sum_k [g_{Mk} P_M Z_{k,i=1}] .$$

We use this approach to produce three types of mutants for each of the 15 different groups of small phytoplankton. These are (i) NO_3 loss mutants, which lose the ability to use nitrate, (ii) NO_3/NO_2 loss mutants, which lose the ability to use both nitrate and nitrite, and (iii) null mutants, which retain the ability to use ammonium, nitrite and nitrate.

For each of the three mutant types, the proportion of replications of the parent population resulting in mutation (Λ_f) was set to a value of $\Lambda_f = 10^{-8}$. Observed mutation rates of microbes with DNA genomes are often approximately 0.003 per genome per replication [6,7]. For picocyanobacteria with genomes of size 1.75 Mbp – 3 Mbp, we would therefore expect mutation rates of approximately 10^{-9} per base pair per replication. Typically, mutations at a number of locations (bases) in the genome can lead to the loss of function of a particular gene or trait. This is called the 'target size' of a mutation. We do not know the target size for the loss of nitrate or nitrite use abilities in picocyanobacteria. We chose a value of Λ_f that is ten-fold greater than the expected mutation rate ($\Lambda_f = 10^{-8} = 10^{-9} \times 10$), corresponding to a target size of approximately 10 bases. It is possible that target sizes for the loss of nitrogen use traits are substantially larger than this value. If so, we can think of the mutants in the model as representing cells with a mutation at a location that is part of a specific subset (*i.e.*, 10 specific bases) of the locations in the mutational target for a phenotype. For instance, these might represent a specific group of mutations that can cause (i) the loss of function of nitrate reductase (NO_3 loss mutant phenotype) (ii) the loss of function of nitrite reductase or (NO_3/NO_2 loss mutant phenotype) and (iii) no change in nitrogen use abilities (null mutant phenotype). Given the considerable uncertainty concerning mutation rates (per base pair) and the target size for nitrogen use targets, we performed additional simulations using a greater mutation rate ($\Lambda_f = 10^{-6}$ see Results, Section 2. *ii*), to make sure our observations were robust to variation in this parameter.

We set the rate of mutations that return mutants to the parent phenotype to a value of $\Gamma_f = 10^{-12}$. It is not clear how often nitrate and nitrite use abilities might be newly acquired by cyanobacteria that lack them, though we anticipate it would be much smaller than the rate at which they are lost. We therefore chose a value that was several orders of magnitude smaller than Λ_f. At this value, Γ_f is so small (and values of P_M remain so small) that is expected to have a negligible affect on the model solutions.

4. Supply of inorganic nutrients

The supply of inorganic nutrients is represented in equation SE1 by the term S_{N_i}, which includes different processes for the inorganic nutrients of different elements. The equations describing the supply of inorganic phosphorus, nitrogen (NO_3, NO_2 and NH_4), iron and silica are as follows:

$$S_{PO4} = r_{DOP} DOP + r_{POP} POP$$

$$S_{Si} = r_{POSi} POSi$$

$$S_{FeT} = r_{DOFe} DOFe + r_{POFe} POFe - c_{scav} Fe' + \alpha F_{atmos}$$

$$S_{NO_3} = \zeta_{NO_3} NO_2$$

$$S_{NO_2} = \zeta_{NO_2} NH_4 - \zeta_{NO_3} NO_2$$

$$S_{NH_4} = r_{DON} DON + r_{PON} PON - \zeta_{NO_2} NH_4$$

Here r_{DOM_i} and r_{POM_i} represent the rate of remineralization of DOM and POM (respectively) forms of the *i*th element. The term c_{scav} is the rate of scavenging of free iron, whose concentration, *Fe'*, is modeled following the approach of Parekh et al. [8]. The term F_{atmos} represents the rate at which iron is deposited at the surface of the ocean in dust, and α represents the solubility of this iron. The oxidation of NO_2 to NO_3 is described by the term ζ_{NO_3}, and the oxidation of NH_4 to NO_2 is described by the term ζ_{NO_2}. In addition, we assume that the oxidation of NH_4 to NO_2 is photoinhibited, such that it does not occur if the ambient light is above 10 μE m^{-2} s^{-1}.

Results

1. Ambient nitrogen concentrations

The annual average concentrations of nitrate, nitrite and ammonium in surface waters (upper 10 m) of the modeled ocean are illustrated in Fig. S2 A, C and E. Annual average concentrations of nitrate, nitrite and ammonium along Atlantic Meridional Transect [9] 13 (AMT 13) in the modeled ocean are illustrated in Fig. S2 D, E and F.

2. Sensitivity of mutant accumulation to model parameters

i. Time dependence of mutant accumulation

In the main text, our analyses concentrate on the abundance of mutants during the fifth year of a model integration (Figs 2 and 3). However, we expect the abundance of mutants in the model to change over time, as they are produced by parent populations. In Fig. S3, the distribution of phytoplankton in surface waters of the global ocean is illustrated for the tenth year of the integration. The distributions of small (parent) and large phytoplankton are similar in the fifth and tenth years. In the tenth year of the integration, mutants have accumulated to greater abundances than in the fifth year, but similar patterns are observed for the relative abundance of NO_3/NO_2 loss mutants and null mutants in different ocean regions. That is, while NO_3/NO_2 loss mutants reach abundances similar to null mutants in topical oligotrophic regions, null mutants are

substantially more abundant than NO_3/NO_2 loss mutants at higher latitudes. This means that the salient observations reported in the main text are not an artifact of the time at which the distribution of the mutants was studied.

ii. Mutation rate
We do not know the mutation rates for the loss of NO_3 use abilities or NO_3/NO_2 use abilities in marine cyanobacteria (see Methods, Section 3). We therefore wanted to study the distribution of mutants in the model using a different value for the mutation rate, and performed a simulation where the proportion of cell divisions resulting in mutations (Λ_f) was set to $\Lambda_f = 10^{-6}$, instead of $\Lambda_f = 10^{-8}$. In the simulation where $\Lambda_f = 10^{-6}$ (Fig. S4) the abundance of NO_3/NO_2 loss mutants and null mutants are approximately two orders of magnitude greater than in the simulation where $\Lambda_f = 10^{-8}$ (Fig. 2). However, changing the parameter Λ_f to 10^{-6} did not substantially change the relative abundance of NO_3/NO_2 loss mutants and null mutants in different ocean regions. This suggests that our observations are robust to variation in the mutation rate over the range of values that were studied.

iii. Oxidation rates of NH_4 and NO_2
We used a model that parameterizes important biogeochemical transformations of nitrogen (see Methods, section 4). There is considerable uncertainty surrounding the rates at which some of these transformations occur in the environment. We therefore performed simulations using different values for two such parameters. Specifically, we used smaller values for the rate of (light-inhibited) oxidation of ammonium to nitrite ($\zeta_{NO_2} = 0.1$ d^{-1}) and the rate of oxidation of nitrite to nitrate ($\zeta_{NO_3} = 0.033$ d^{-1}). In Fig. S5, ambient concentrations of nitrate, nitrite and ammonium are illustrated for this integration, and in Fig. S6, the distribution of phytoplankton is illustrated for this integration. The lower values of ζ_{NO_2} and ζ_{NO_3} result in small changes in the distribution of ammonium and nitrite, including greater concentrations of ammonium in the lower euphotic zone, and near the surface in several regions with high productivity. However, changing the rates of oxidation of NH_4 and NO_2 did not substantially alter the observation that NO_3/NO_2 loss mutants reach similar abundances to null mutants in topical oligotrophic regions, but at higher latitudes, null mutants reach much higher abundances than NO_3/NO_2 loss mutants.

iv. Randomly generated phytoplankton communities
We performed integrations of the model with 10 randomly generated phytoplankton communities. In the main text, results are presented for one of these communities. In Fig. S7, the distribution of phytoplankton in global surface waters is illustrated for the remaining nine phytoplankton communities. There is some variation among these integrations, for example in the dominance of particular regions by large versus small phytoplankton. However, some important features of the distribution of NO_3/NO_2 loss mutants and null mutants are common to each of these integrations. In each case, NO_3/NO_2 loss mutants accumulate to similar abundances to null mutants in tropical oligotrophic regions, but at higher latitudes, null mutants occur at substantially greater abundances that NO_3/NO_2 loss mutants, indicating that NO_3/NO_2 loss mutants are disadvantaged in these regions.

Table S1. Parameters used in the model.

parameter	description	value	units
μ	Maximum phytoplankton growth rate	Small: 1.4<br>Large: 2.5	d^{-1}
m^p	Phytoplankton mortality rate	Small: 0.1<br>Large: 0.1	d^{-1}
k_{PAR}	PAR saturation coefficient	Small: mean 0.012, std 0.02<br>Large: mean 0.012, std 0.006<br>(absolute value)	$(\mu E\ m^{-2}\ s^{-1})^{-1}$
k_{inhib}	PAR inhibition coefficient	Small: mean 6×10^{-3}, std 1×10^{-4}<br>Large: mean 1×10^{-3}, std 5×10^{-5}<br>(absolute value)	$(\mu E\ m^{-2}\ s^{-1})^{-1}$
A	Temperature curve coefficient	1.04	
T_o	Temperature optimum coefficient	-2 to 30	^oC
B	Temperature range coefficient	Small: 1×10^{-3}<br>Large: 3×10^{-4}	$^oC^{-1}$
C	Temperature decay coefficient	4	
τ_1, τ_2	Temperature normalization coefficients	3, 0.3	
K_{PO4}	Phosphate half saturation	Small: 1.0×10^{-2} to 1.5×10^{-2}<br>Large: 3.5×10^{-2} to 5.5×10^{-2}	µM P
K_{NO3}	Nitrate half saturation	Small: 0.16 to 0.24<br>Large: 0.56 to 0.88	µM N
K_{NO2}	Nitrite half saturation	Small: 0.16 to 0.24<br>Large: 0.56 to 0.88	µM N
K_{NH4}	Ammonium half saturation	Small: 0.08 to 0.12<br>Large: 0.28 to to 0.44	µM N
K_{si}	Silicic acid half saturation	Non-diatom: 0<br>Diatom: 1	µM Si
K_{Fe}	Iron half saturation	Small: 1×10^{-5} to 1.5×10^{-5}<br>Large: 3.5×10^{-5} to 5.5×10^{-5}	µM Fe
$R_{Si:P}$<br>$R_{N:P}$<br>$R_{Fe:P}$	Phytoplankton elemental ratios	16<br>16<br>1×10^{-3}	
ψ	Ammonia/nitrite inhibition	4.6	$(\mu M\ N)^{-1}$
w^p	Phytoplankton sinking rate	Small: 0<br>Large: 0.5	$m\ d^{-1}$
f_{POM}	Decomposing material – proportion to POM	Small phytoplankton: 0.2<br>Large phytoplankton: 0.5<br>Small zooplankton: 0.2<br>Large zooplankton: 0.7	
g_{POM}	Grazed, unconsumed material – proportion to POM	0.8	
g_m	Zooplankton maximum grazing rate	0.5	d^{-1}

parameter	description	value	units
η	Palatability of phytoplankton to zooplankton	Large phyto, large zoo: 1<br>Small phyto, small zoo: 1<br>Large phyto, small zoo: 0.2<br>Small phyto, large zoo: 0.2<br>Diatom, large zoo: 0.7<br>Diatom, small zoo: 0.14	
m^z	Zooplankton mortality rate	0.033	d^{-1}
k^p	Phytoplankton half saturation	0.025	μM P
r_{DOP}	DOM remineralization rate	0.01	d^{-1}
r_{DON}		0.01	
r_{DOFe}		0.01	
r_{POP}	POM remineralization rate	0.02	d^{-1}
r_{PON}		0.02	
r_{POFe}		0.02	
r_{POSi}		3.3×10^{-3}	
w_{POM}	POM sinking rate	10	$m\ d^{-1}$
ζ_{NO2}	NH_4 to NO_2 oxidation rate	2	d^{-1}
ζ_{NO3}	NO_2 to NO_3 oxidation rate	0.1	d^{-1}
α_{Fe}	Iron solubility constant	0.04	
c_{Fe}	Iron scavenging rate	1.1×10^{-3}	d^{-1}
β_{Fe}	Ligand binding strength	2×10^{5}	$(\mu M\ Fe)^{-1}$
k_o	PAR attenuation coefficient	0.04	m^{-1}
k_{phyto}	PAR attenuation coefficient from phytoplankton	0.64	$(\mu M\ P)^{-1}\ m^{-1}$

Supplementary References

1. Follows MJ, Dutkiewicz S, Grant S, Chisholm SW (2007) Emergent biogeography of microbial communities in a model ocean. Science 315: 1843-1846.
2. Dutkiewicz S, Follows MJ, Bragg JG (2009) Modeling the coupling of ocean ecology and biogeochemistry. Global Biogeochemical Cycles 23: GB4017.
3. Wunsch C, Heimbach P (2006) Practical global state estimation. Physica D 230: 197-208.
4. Marshall J, Hill C, Perelman L, Adcroft A (1997) Hydrostatic, quasi-hydrostatic, and non-hydrostatic ocean modeling. Journal of Geophysical Research 102: 5733-5752.
5. Roe PL (1985) Some contributions to the modelling of discontinuous flows. Proc. 1983 AMS-SIAM Summer Seminar on Large Scale Computing in Fluid Mechanics. Lectures in Applied Mathematics 22: 163-193.
6. Drake JW (1991) A constant rate of spontaneous mutation in DNA-based microbes. Proceedings of the National Academy of Sciences of the United States of America 88: 7160-7164.
7. Drake JW, Charlesworth B, Charlesworth D, Crow JF (1998) Rates of spontaneous mutation. Genetics 148: 1667-1686.
8. Parekh P, Follows MJ, Boyle E (2004) Modeling the global ocean iron cycle. Global Biogeochemical Cycles 18: GB1002.
9. Aiken J, Rees N, Hooker S, Holligan P, Bale A, et al. (2000) The Atlantic Meridional Transect: overview and synthesis of data. Progress in Oceanography 45: 257-312.

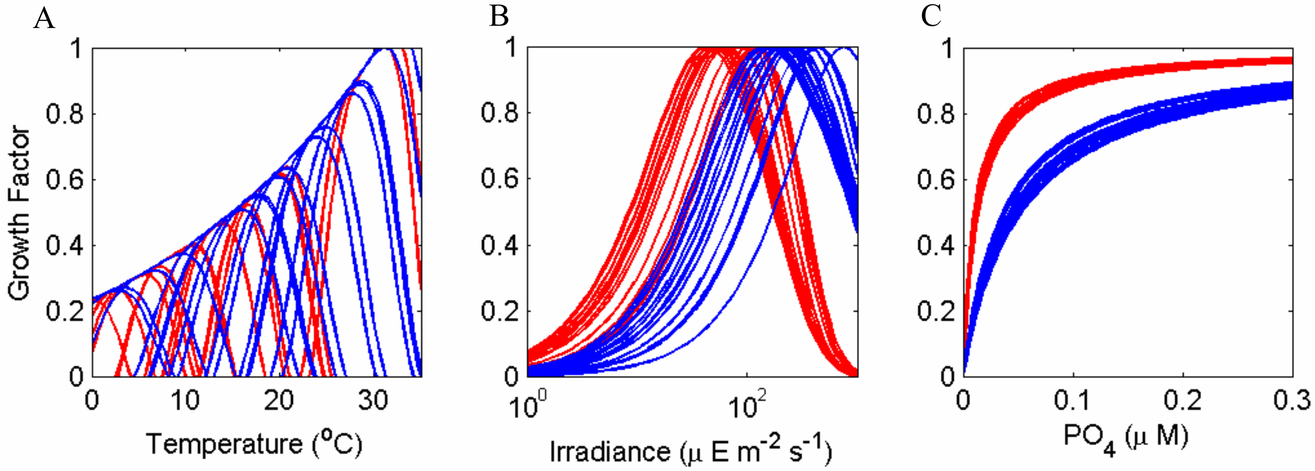

Fig. S1. Growth of modeled phytoplankton as a function of (A) temperature (B) irradiance and (C) ambient phosphorus concentration. Here growth curves are illustrated for the 15 small phytoplankton types (red) and 18 large phytoplankton types (blue) from the simulation presented in the main text.

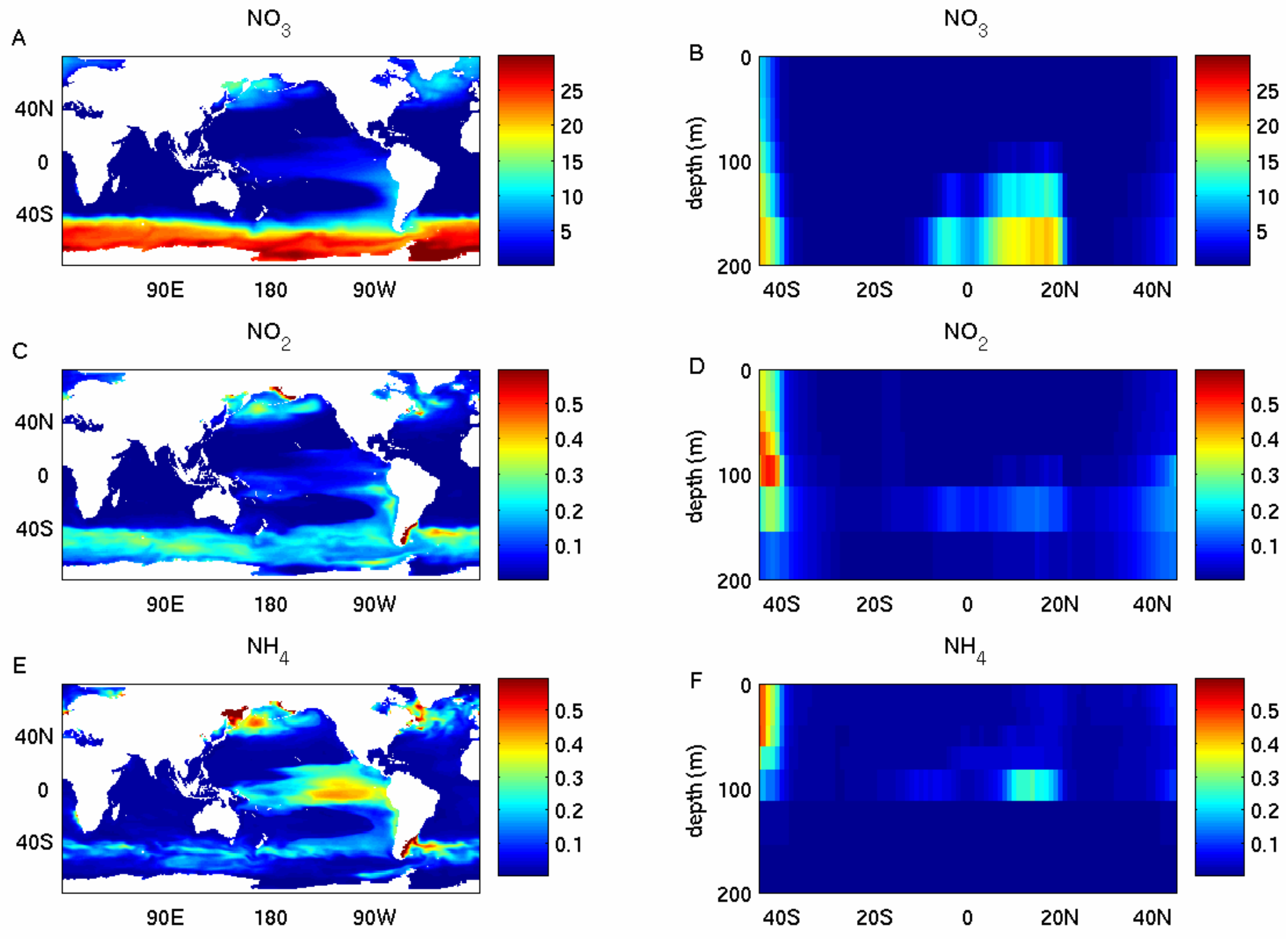

Fig. S2. Ambient availability of nitrogen. Annual average concentrations (μ M) of nitrate (A, B), nitrite (C, D) and ammonium (E, F) in surface waters of the global ocean (0-10 m; A, C, E), and along a transect in the Atlantic Ocean (AMT 13; B, D, F).

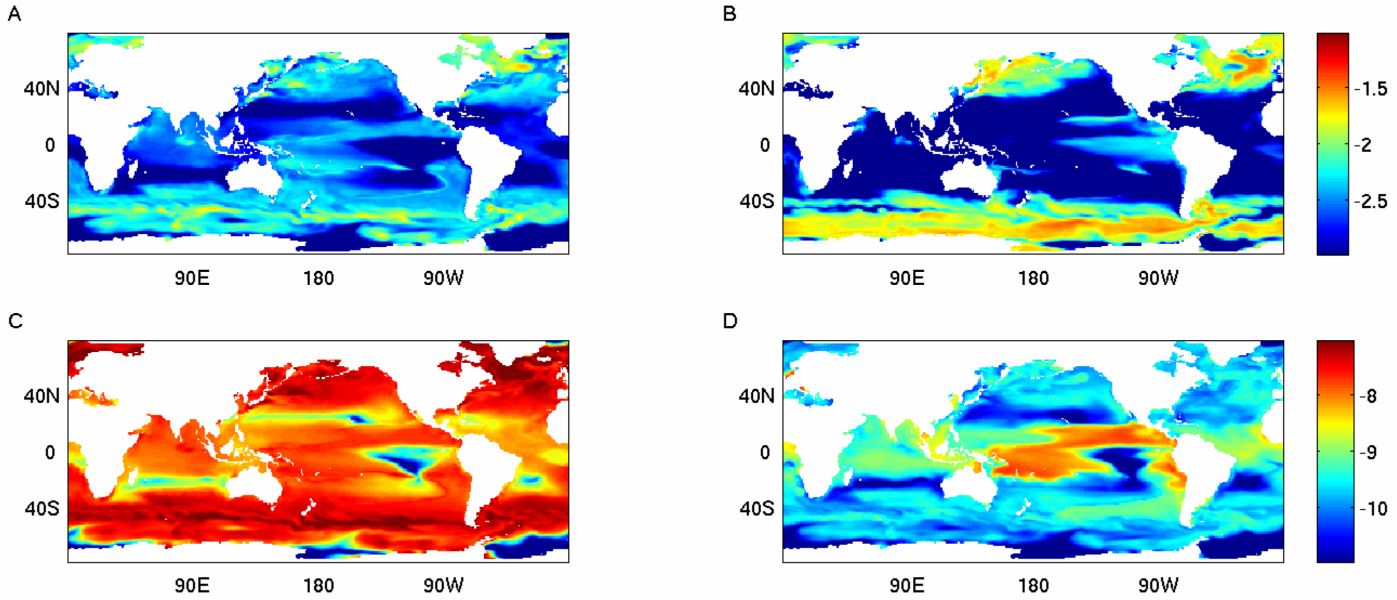

Fig. S3. The distribution of phytoplankton in surface waters of the model ocean during the tenth year of a model integration. Phytoplankton abundance (biomass, in units of μM P, 0 to 10 M, annual average) is plotted for (A) picophytoplankton and (B) large phytoplankton. The distribution of mutant picophytoplankton (biomass in units of μM P, 0 to 10 m, annual average) is plotted for (C) null mutants and (D) NO_3/NO_2 loss mutants.

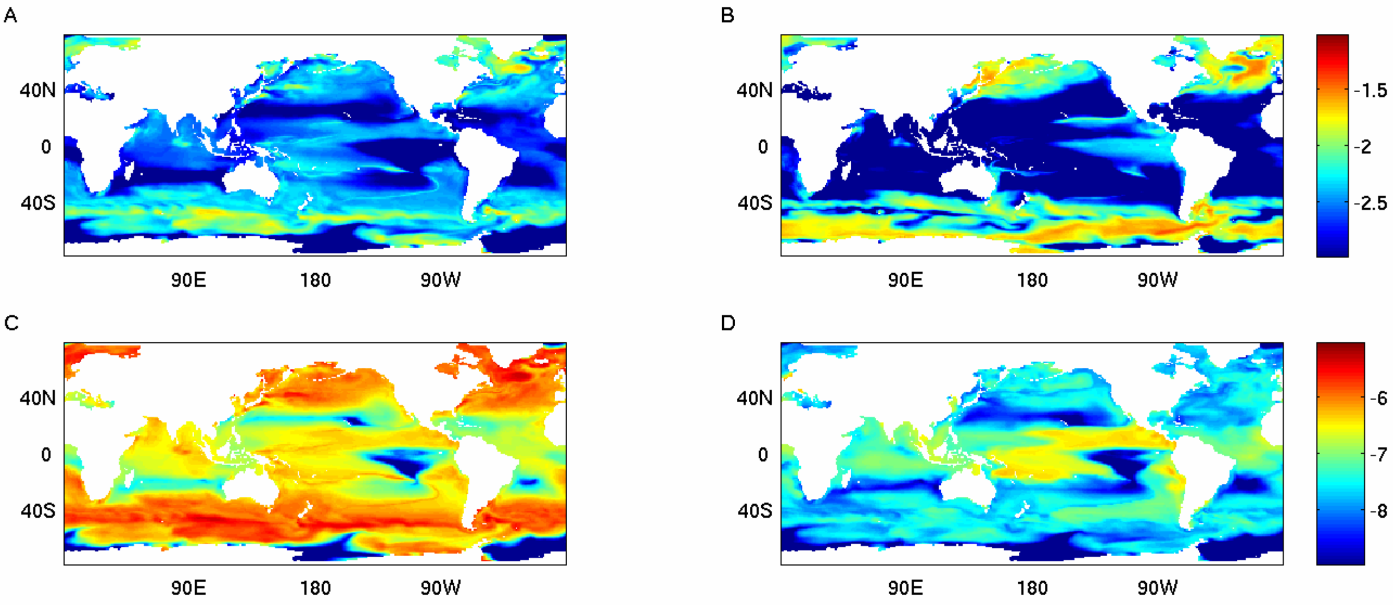

Fig. S4. The distribution of phytoplankton in surface waters of the model ocean during the fifth year of a model integration, with the proportion of cell divisions resulting in mutations (Λ_f) set to $\Lambda_f = 10^{-6}$. Phytoplankton abundance (biomass, in units of μM P, 0 to 10 M, annual average) is plotted for (A) picophytoplankton and (B) large phytoplankton. The distribution of mutant picophytoplankton (biomass in units of μM P, 0 to 10 m, annual average) is plotted for (C) null mutants and (D) NO_3/NO_2 loss mutants.

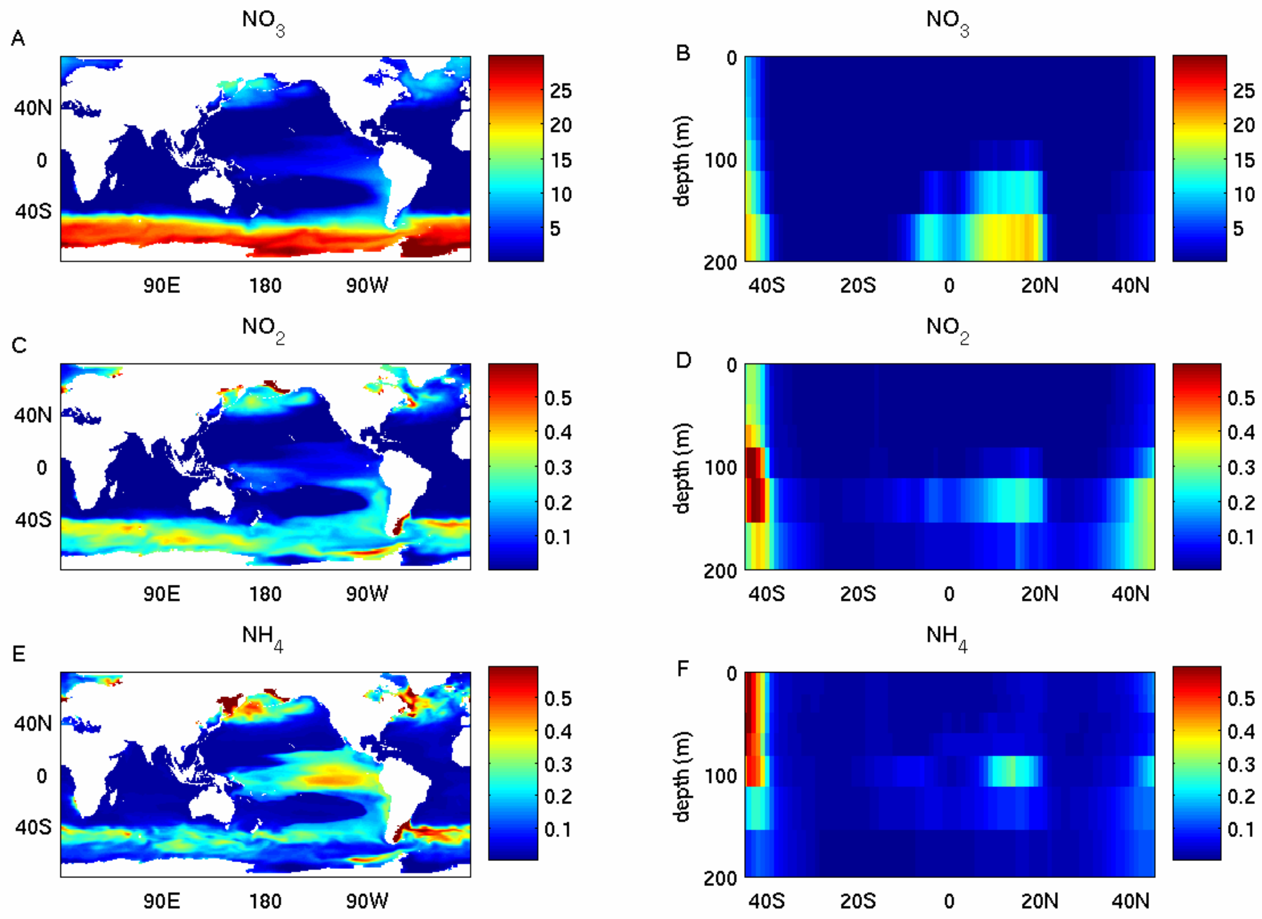

Fig. S5. Ambient availability of nitrogen in an integration with ζ_{NO_2} = 0.1 d^{-1} and ζ_{NO_3} = 0.033 d^{-1}. Annual average concentrations (μ M) of nitrate (A, B), nitrite (C, D) and ammonium (E, F) in surface waters of the global ocean (0-10 m; A, C, E), and along a transect in the Atlantic Ocean (AMT 13; B, D, F).

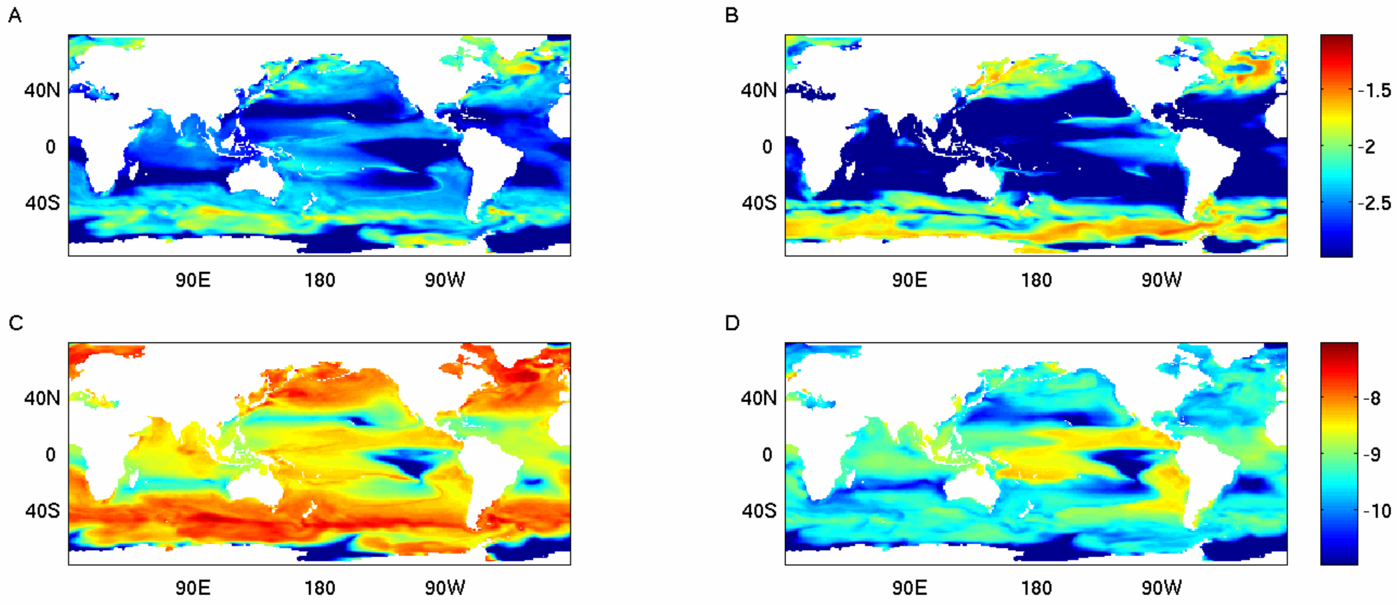

Fig. S6. The distribution of phytoplankton in surface waters of the model ocean during the fifth year of a model integration with $\zeta_{NO_2} = 0.1$ d^{-1} and $\zeta_{NO_3} = 0.033$ d^{-1}. Phytoplankton abundance (biomass, in units of µM P, 0 to 10 M, annual average) is plotted for (A) picophytoplankton and (B) large phytoplankton. The distribution of mutant picophytoplankton (biomass in units of µM P, 0 to 10 m, annual average) is plotted for (C) null mutants and (D) NO_3/NO_2 loss mutants.

integration ii

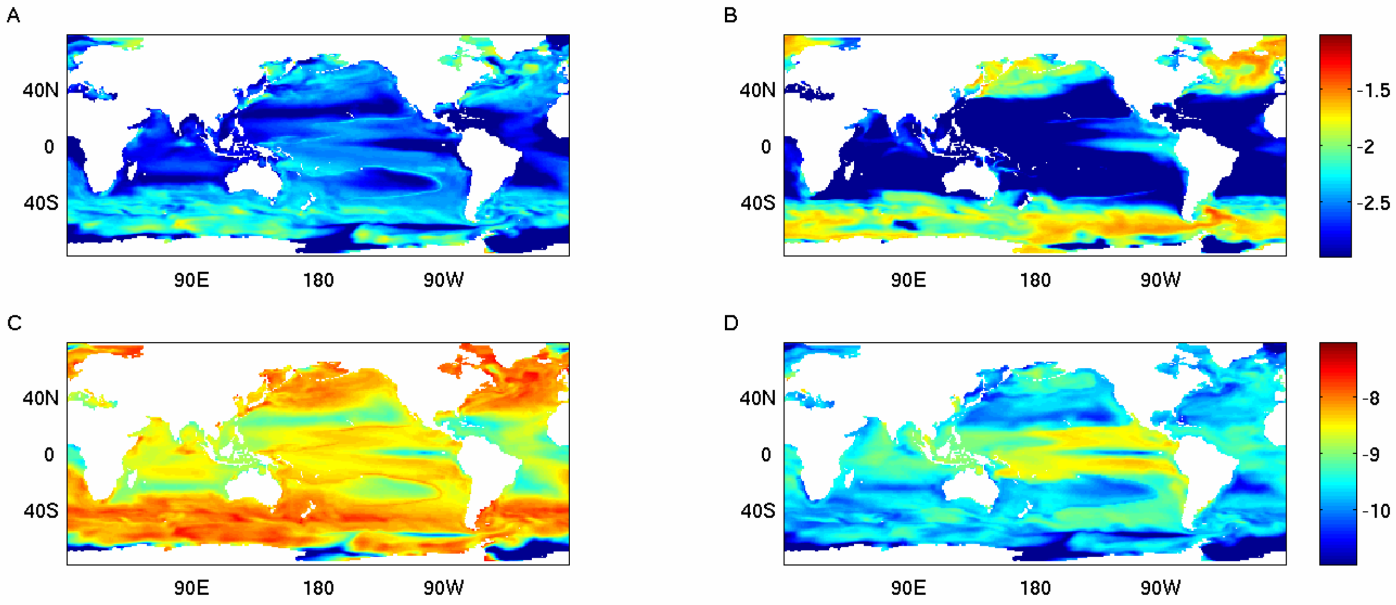

Fig. S7. The distribution of phytoplankton in surface waters of the model ocean during the fifth year of nine additional model integrations (integrations ii-x) using different, randomly generated, phytoplankton communities. Phytoplankton abundance (biomass, in units of µM P, 0 to 10 M, annual average) is plotted for (A) picophytoplankton and (B) large phytoplankton. The distribution of mutant picophytoplankton (biomass in units of µM P, 0 to 10 m, annual average) is plotted for (C) null mutants and (D) NO_3/NO_2 loss mutants.

integration iii

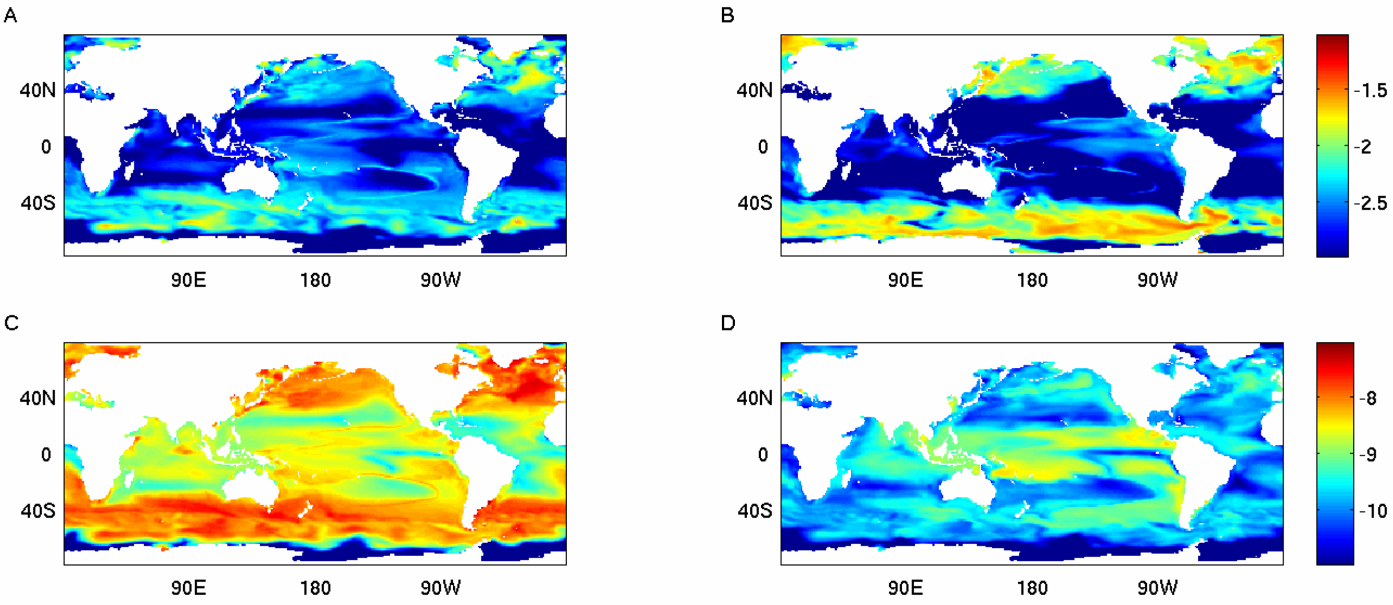

integration iv

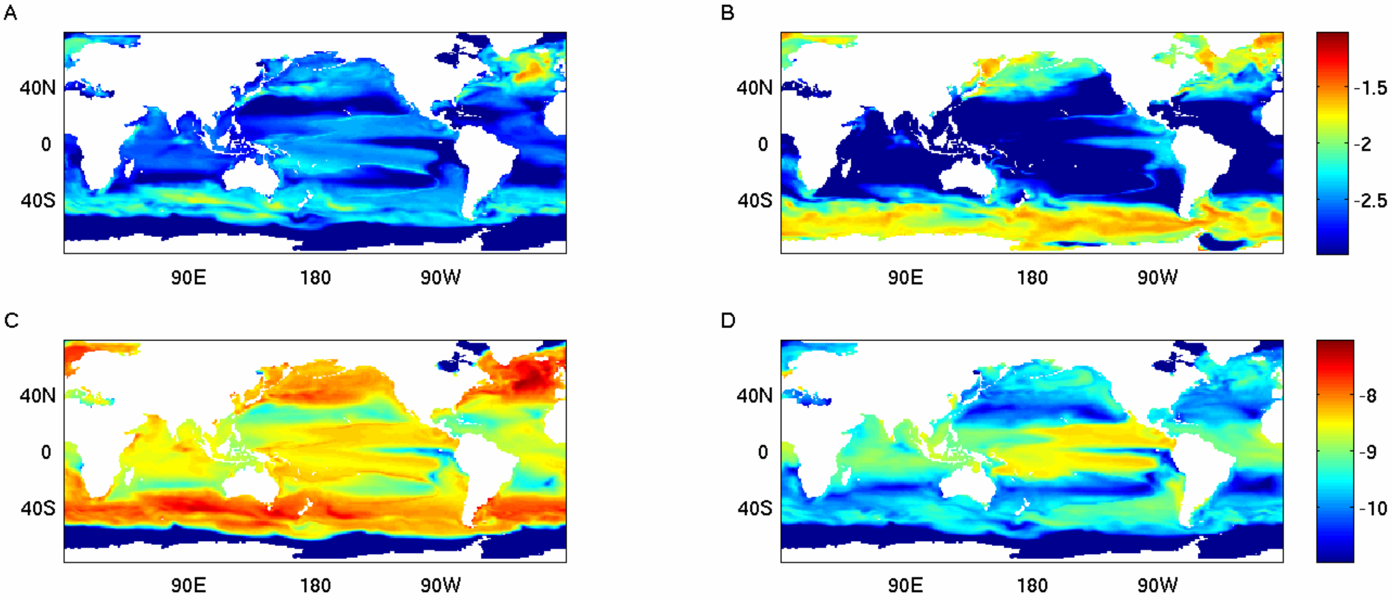

Fig. S7. continued.

integration v

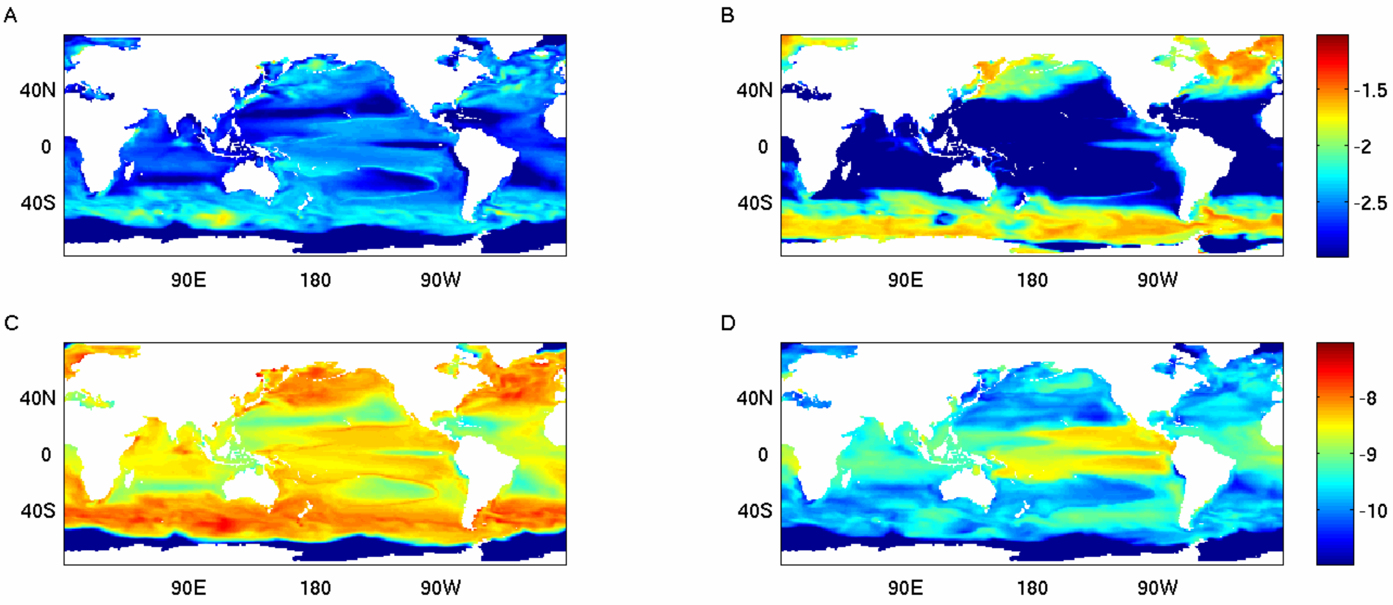

integration vi

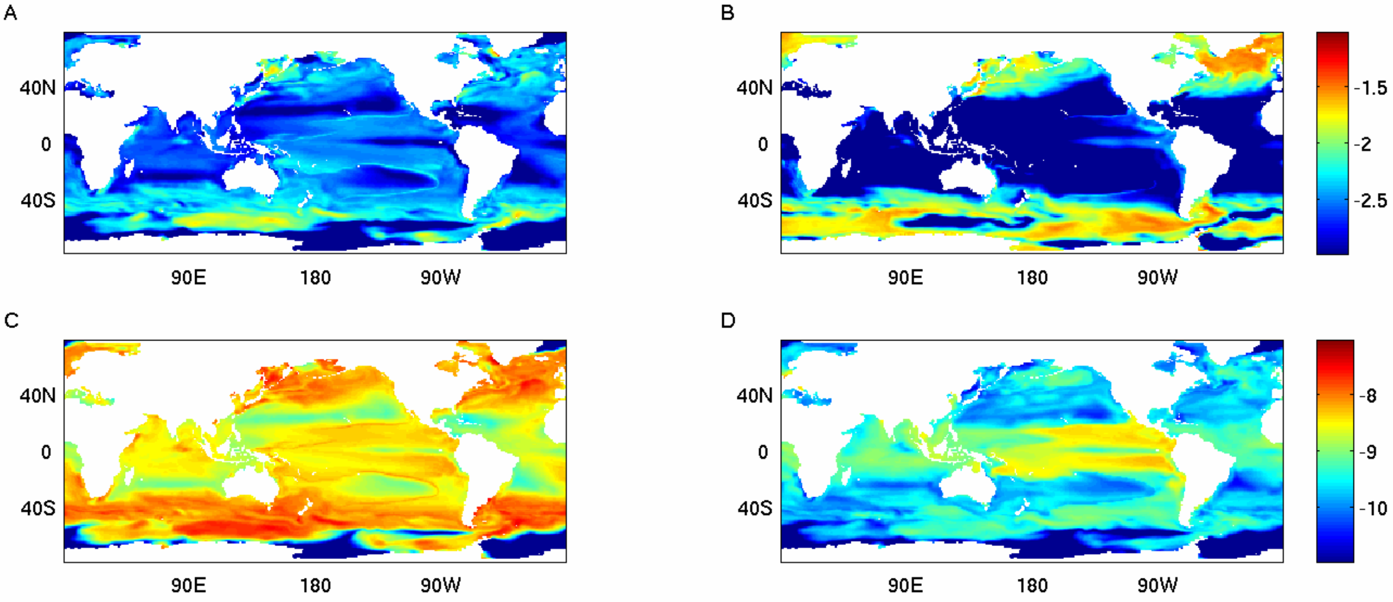

Fig. S7. continued.

integration vii

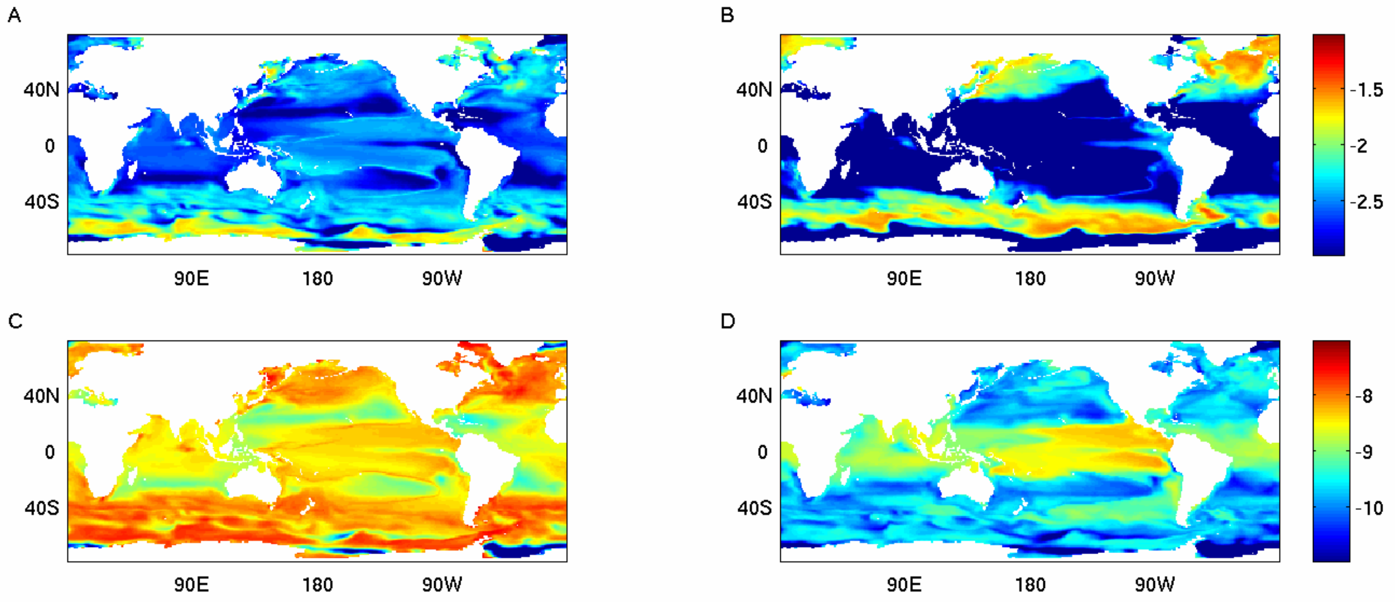

integration viii

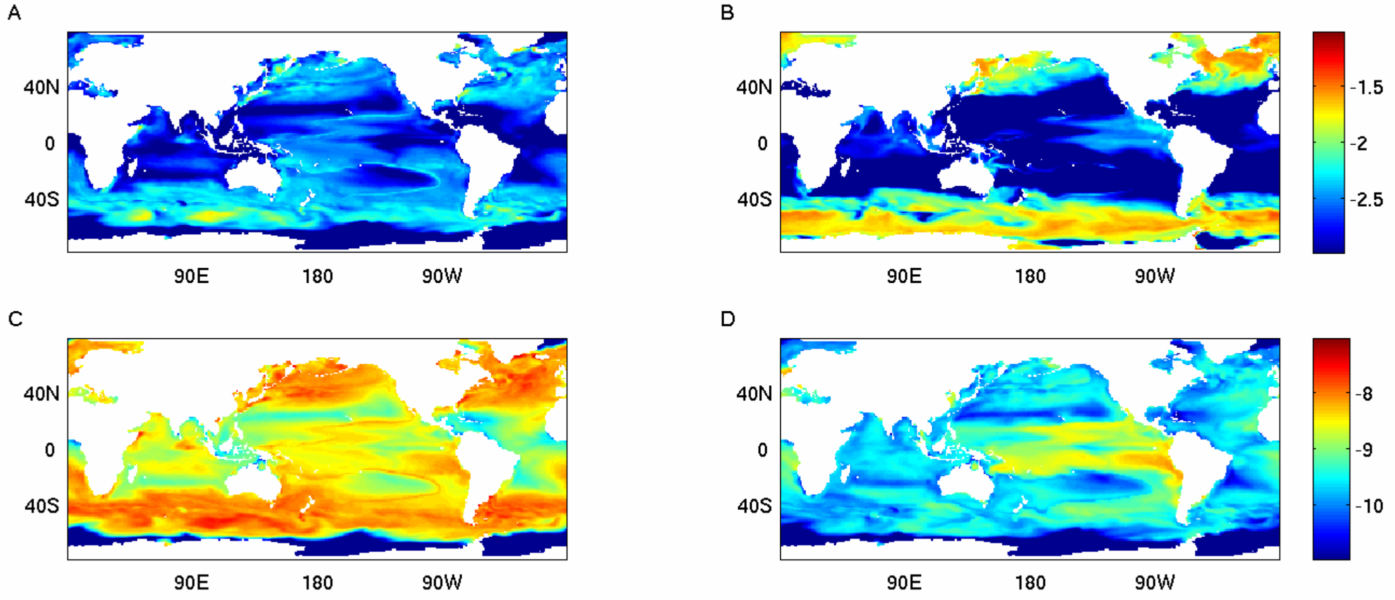

Fig. S7. continued.

integration ix

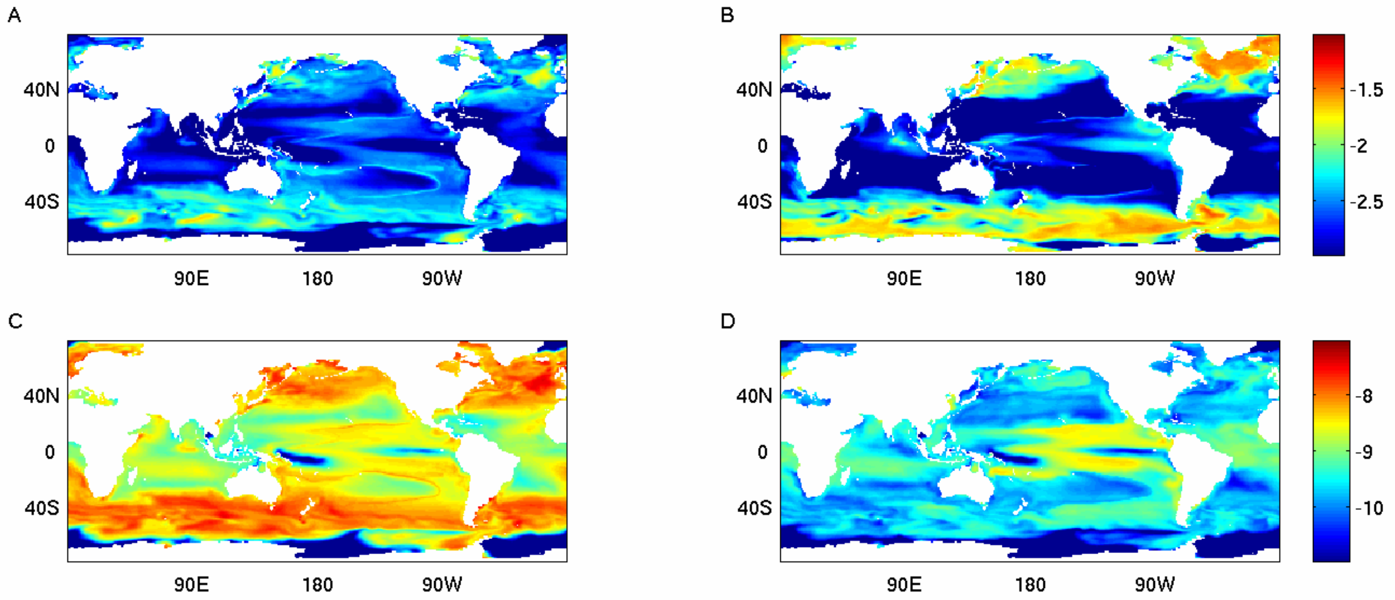

integration x

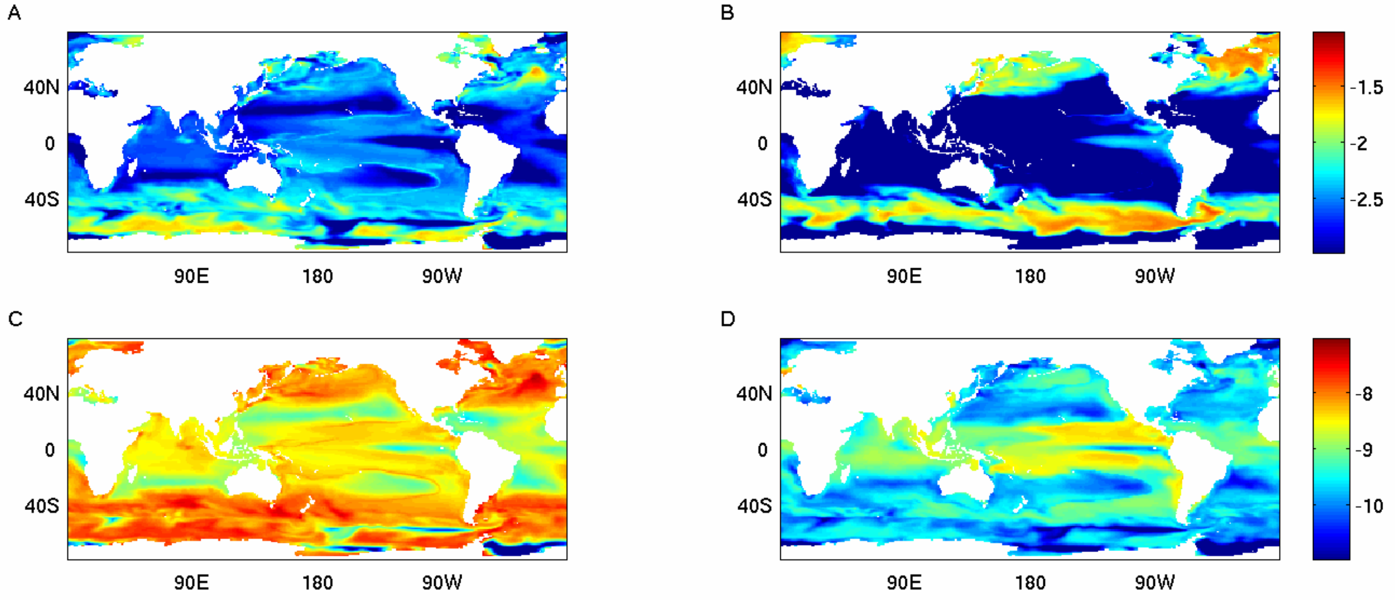

Fig. S7. continued.
